# Mutational signatures association with replication timing in normal cells reveals similarities and differences with matched cancer tissues

**DOI:** 10.1038/s41598-023-34631-9

**Published:** 2023-05-15

**Authors:** Adar Yaacov, Shai Rosenberg, Itamar Simon

**Affiliations:** 1 Gaffin Center for Neuro-Oncology, Sharett Institute for Oncology, Hadassah-Hebrew University Medical Center, Jerusalem, Israel; 2 The Wohl Institute for Translational Medicine, Hadassah-Hebrew University Medical Center, Jerusalem, Israel; 3 Department of Microbiology and Molecular Genetics, IMRIC, Faculty of Medicine, Hebrew University of Jerusalem, Jerusalem, Israel

**Keywords:** Cancer genomics, Computational biology and bioinformatics, DNA replication, DNA damage and repair

## Abstract

Mutational signatures’ association with replication timing (RT) has been studied in cancer samples, but the RT distribution of somatic mutations in non-cancerous cells was only minimally explored. Here, we performed comprehensive analyses of mutational signatures in 2.9 million somatic mutations across multiple non-cancerous tissues, stratified by early and late RT regions. We found that many mutational processes are active mainly or solely in early RT, such as SBS16 in hepatocytes and SBS88 in the colon, or in late RT, such as SBS4 in lung and hepatocytes, and SBS18 across many tissues. The two ubiquitous signatures, SBS1 and SBS5, showed late and early bias, respectively, across multiple tissues and in mutations representing germ cells. We also performed a direct comparison with cancer samples in 4 matched tissue-cancer types. Unexpectedly, while for most signatures the RT bias was consistent in normal tissue and in cancer, we found that SBS1’s late RT bias is lost in cancer.

## Introduction

The process of DNA replication plays an important role in mutagenesis^1^, as mismatches can be introduced, and DNA damage may be fixed into mutations during DNA replication. Indeed, several replication features (such as fork rate and direction) are known to be associated with certain types of mutation loads^2,3^. Moreover, replication timing (RT), the relative time in S phase that each genomic region is replicated^4^, is found to be associated with mutation load. The RT of a region reflects a higher order of genomic organization as it correlates with basic chromosomal features such as the regional GC content, Giemsa banding, gene density, chromatin accessibility and transcription^5^. RT is strongly associated with mutation rates of both germline and somatic cells, which are much higher in genomic regions that replicate later in S phase (reviewed in Ref.^6^), suggesting that either mutagenesis or repair occurs in different intensities in early and late replicating regions.

Somatic mutations in cancer genomes are accumulated along all stages of the cell lineage and are the summation of multiple mutational processes^7^. Different mutational processes generate unique combinations of mutation types, termed “Mutational Signatures”. Analysis of mutation frequency revealed many mutational signatures that are indicative of various mutagenesis processes^8^. Some of the signatures are found in all tumor types, indicating that they stem from a very general mutagenesis process, whereas others are characteristic of a single type of cancer due to a tissue-specific mutagenesis process.

Recently, we have studied the association of various mutational processes with RT by systematically analyzing the mutational landscape of 2787 WGS tumors from 32 tumor types, separately for early and late replicating regions (ERR and LRR). We found that many mutational processes are associated with RT. However, the associations are signature specific: some signatures are associated with early or late replication (such as SBS7b and SBS7a, respectively), while others have no association^9^. The mechanistic basis of the association between RT and mutability is not fully understood. Partial explanation is a differential activity of key DNA repair pathways in ERR and LRR, which differ in their chromatin organization. Indeed, tumors with defects in either mismatch repair (MMR) or global genome nucleotide excision repair (GG-NER) mechanisms do not show higher mutation rates in LRR^10,11^.

Cancer transformation causes major changes in the transformed cells, including an increase in the mutation rates^12^, and changes in the chromatin environment^13^. Such changes may affect the association between mutability and RT; thus, it is important to explore the association between the various mutational processes and RT in normal tissues and to compare it to the associations observed in transformed cells^9^. Recently, there is accumulative information on somatic mutations in normal tissues, mainly based on WGS data. The SomaMutDB database compiled 2.42 million SNVs and 0.12 million INDELs identified in nineteen normal tissues and cell types reported, using 2838 single cells, clones or biopsies from 374 human subjects^14^. In addition, a recent paper sequenced the entire genome (30 × average coverage) of 389 patches of 29 distinct histological structures from multiple samples from the same individuals and depicted the landscape of somatic and germline mutations^15^. Although these data allow exploring the association between RT and mutability in normal somatic tissues, as well as exploring the differences between somatic and germline mutations, the RT bias of mutational signatures in non-cancerous cells has been assessed only to a minimal extent^15^.

Here we took advantage of the increasing data on somatic mutation in normal tissues and analyzed the association of such mutations to RT using the methodology we have established^9^. We found that mutational signatures have a consistent RT bias in non-cancerous cells across cohorts, and that the bias mostly correlates with the bias seen in matching cancerous cells. The major exception is SBS1, a ubiquitous, clock-wise mutational signature stemming from failure to repair G:T mismatches initiated by spontaneous or enzymatic deamination of 5-methylcytosine to thymine. SBS1 was highly LRR biased in non-cancerous tissues, while this bias is lost in cancer. For other signatures, our analyses demonstrated that most mutational processes appear in normal cells with the same basic features.

## Results

### Replication timing bias of mutational signatures in non-cancerous cells

**Figure 1:**
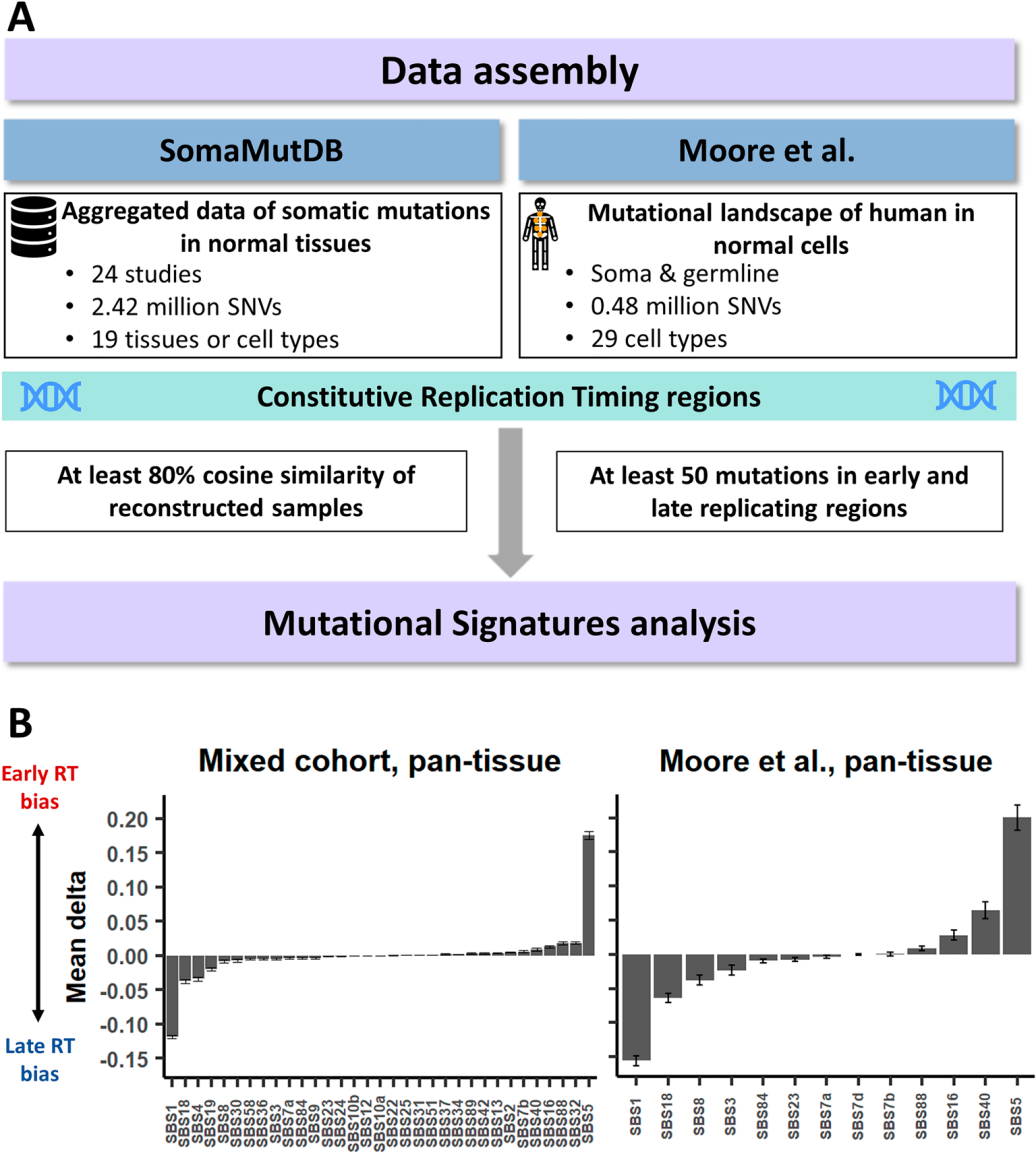
Workflow and overview. (**A**) Workflow, including input, methodology, and inclusion criteria. (**B**) Mean RT bias delta. X axis, SBS signatures which are found in the cohorts (left, mixed cohort of 15 studies; right, Moore et al. study). Y axis, delta between relative contribution of signatures in ERR and LRR. Positive value implies higher contribution in ERR; negative value implies higher contribution in LRR. Error bars represent standard error of the mean.

We analyzed data from 25 different published papers on mutagenesis in non-cancerous cells (Fig. 1A). First, we composed two independent pan-tissue datasets: (i) “Mixed cohort”: Somatic mutations from 24 papers, most containing data on 1–3 tissues each, retrieved from SomaMutDB^14^, and (ii) Somatic mutations from Moore et al.^15^, which explored somatic mutagenesis in a pan-tissue manner. Then, we explored the mutational landscape following the methodology we have recently established to identify dependency between mutational signatures and RT^9^. Briefly, we limited our analyses to constitutive early and late replication timing regions (ERR and LRR, respectively), which were constructed by the replication timing (RT) profiles of 26 different tissues (“Methods”). These RT regions, constitutes approximately 40% of the genome, were shown to be robust in terms of RT across different tissues and in terms of RT in cancer models (“Methods”). By analyzing mutational signatures separately in ERR and LRR, we can explore which signatures are biased towards a specific RT region (see “Methods”) In total, 1192 and 176 samples from Mixed cohort and Moore et al., respectively, passed the inclusion criteria for mutational signatures and downstream analyses (at least 50 mutations both in ERR and in LRR; cosine similarity of at least 80% of the tumor mutational profile and the reconstructed profile; see “Methods”). The differences (delta) between each mutational signature’s relative contribution in ERR and LRR (after correcting for trinucleotide context differences between the regions, see “Methods”) were calculated. The results were highly similar between the Mixed cohort and the Moore et al. cohort (R = 0.967, P < 3e^−7^, Pearson’s correlation; Fig. 1B). Therefore, we united them into a one bigger cohort for further analyses. Moreover, to make sure the results are not highly influenced by the specific mutational signatures framework, we performed similar analysis using deconstructSigs algorithm^16^ and found highly similar results (R = 0.974, P < 5e^−7^, Pearson’s correlation; Supplementary Fig. 1).

**Figure 2:**
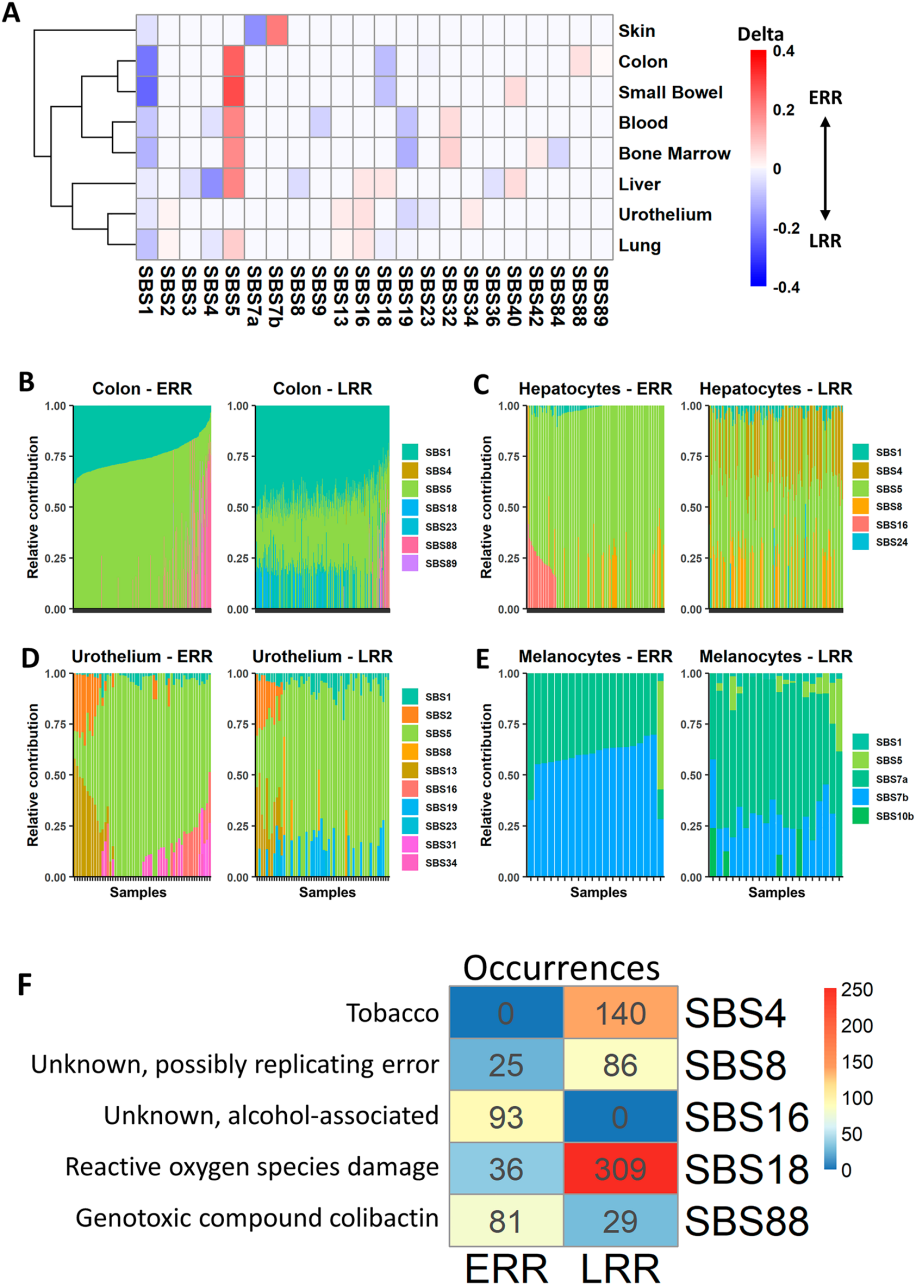
Replication timing bias of mutational signatures in non-cancer tissues. (**A**) Heatmap showing each SBS signature’s RT bias (P < 0.05, FDR corrected Wilcoxon rank sum test), or no bias (white), stratified by tissues. Red and blue indicate positive and negative delta respectively. Only tissues with at least 15 samples are presented. Signatures with no bias in any project are not shown. (**B**–**E**) Stacked bar plots showing the proportions of contribution of each SBS signature in different tissues in ERR and LRR (left and right in each tissue-specific plot, respectively). Each bar represents one sample. The order of the samples is the same in ERR and LRR panels. (**B**) Colon; (**C**) Urothelium; (**D**) hepatocytes; (**E**) melanocytes. In each tissue type, samples (X axis) are ordered the same in ERR and LRR. (**F**) Occurrences of SBS signatures in non-cancer tissues as extracted by the SigProfilerExtractor algorithm across all analyzed samples. The colors indicate the number of occurrences, from 0 (blue) to the highest (red).

The two most common signatures in non-cancerous tissues are SBS1 and SBS5^15^. Interestingly, these two signatures showed the greatest mean RT delta—SBS5 had a bias towards ERR, and SBS1 towards LRR, both in a pan-tissue analysis and across most tissues in individual tissue analyses (Figs. 1B, 2A, Supplementary Fig. 2). This is due to (i) consistent RT bias across cancers and (ii) the signatures’ prevalence. Other signatures were also consistent in their RT bias across cohorts and across tissues: SBS7b, SBS16, SBS40 and SBS88 were biased to ERR, while SBS4, SBS7a, SBS8 and SBS18 were biased towards LRR (Figs. 1B, 2A–F).

Of note is the strong association of the relatively new signature, SBS88, with ERR. This signature is associated with genotoxic compound colibactin produced by *E. coli* bacteria carrying pks pathogenicity island^17^ and we found that it is more abundant in ERR (Fig. 2A,B,F). To our knowledge, this is the first assessment of SBS88’s RT bias. Several signatures showed RT bias only in certain tissues, for example, APOBEC-related signatures SBS2 and SBS13 were ERR biased in lung and urothelium, while SBS9 and SBS84 showed LRR bias in blood cells and in bone marrow, respectively (Fig. 2A–E).

Usually, RT biases stem from differences in the magnitude of the contribution of the signatures in ERR and LRR (e.g., SBS1, SBS5, SBS2/13, SBS7a/b). However, there are extreme cases in which the signatures have exposure only in ERR or in LRR. This occurs, for example, in SBS4, associated with tobacco use, which appeared in 140 samples in LRR and not once in ERR, and in SBS16, which its etiology is unknown, that appeared in 75 samples in ERR and not once in LRR (Fig. 2F).

### Comparison of mutational signatures RT bias in non-cancerous tissues to cancerous tissues

**Figure 3:**
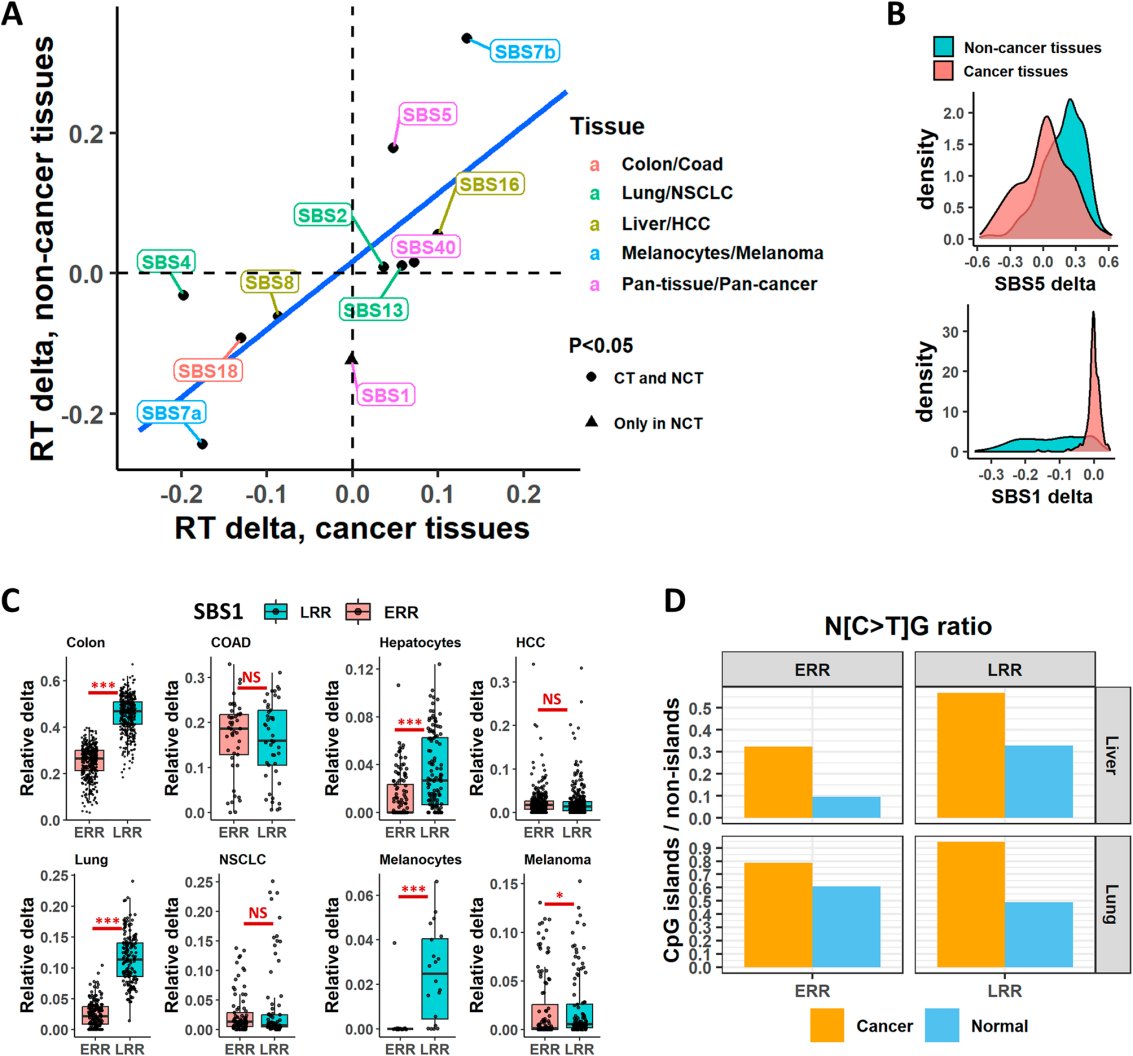
Comparison of mutational signatures RT bias between cancer and non-cancer tissues. (**A**) Mean delta in cancer (X axis) and non-cancer (Y axis) tissues for tissue/cancer-specific and pan-tissue/cancer signatures extracted in both datasets. Colors of labels indicate tissue and matching cancer type for each signature analyzed. Note that SBS1, SBS5 and SBS40 are considered pan-cancer signature and thus included in a pan-tissue/pan-cancer manner. Shape of each point indicates whether the RT associations were statistically significant (P < 0.05, FDR-corrected Wilcoxon rank-sum test) in both datasets (i.e., both in normal and cancer cells; dot) or only in non-cancer tissues (triangle). The latter statistical tests were performed in each setting (tissue/cancer and signature) independently. *CT* cancer tissues. *NCT* non-cancer tissues. (**B**) Density plots showing the distribution of RT delta of SBS5 (upper) and SBS1 (lower) across all samples, stratified by cancer and non-cancer tissues. Value of ~ 0 in X axis means no RT bias. (**C**) Boxplots showing the contribution of SBS1 in ERR and LRR in a normal tissue compared to a matched cancer tissue. Left upper, non-cancer colon cells (left) and colon adenocarcinoma samples (right); Right upper, hepatocytes (left) and Hepatocellular carcinoma (right); Left lower, Lung (left) and NSCLC (right); Right lower, melanocytes (left) and melanoma (right). NSCLC, non-small cell lung cancer. *P < 0.05; **P < 0.001; ***P < 0.0001. (**D**) Barplots showing the ratio of N[C > T]G mutations frequencies in CpG islands and non-islands regions (see “Methods”).

Several studies have investigated the RT bias of mutational signatures in cancer^2,3,9,18–20^ However, to our knowledge, we are the first to study this phenomenon in non-cancerous tissues. Thus, we sought to compare the bias in those tissues. Interestingly, the results were mostly similar to our recently published paper^9^. For example, the ERR biases of SBS5, SBS7b and SBS16, and the LRR biases of SBS4, SBS7a, SBS8 and SBS18. However, due to difference in methodology of mutational signatures identification between Yaacov et al. and in this work, we re-analyzed the RT bias of signatures in 4 cancer projects from the Pan-Caner Analysis of Whole Genomes (PCAWG)^21^, using the same methodology as we analyzed the non-cancerous tissue (“Methods”), and for which we had a matched tissue with non-cancerous samples: Colon and Colon adenocarcinoma (COAD); Hepatocytes and Hepatocellular carcinoma (HCC); Lung and Non-small cell lung cancer (NSCLC); and Melanocytes and Melanoma. For tissue-specific signatures SBS2, SBS4, SBS7a/b, SBS8, SBS13, SBS16, and SBS18, we examined the bias in a tissue-specific manner, while for signatures SBS1, SBS5 and SBS40, which are considered pan-cancer signatures, we examined the bias in a pan-tissue/pan-cancer approach^22^. Overall, most signatures had the same bias direction (ERR or LRR) (Fig. 3A), including the pan-tissue/pan-cancer signatures SBS5 and SBS40; the UV-related SBS7a/b, the APOBEC-related SBS2 and SBS13, the tobacco-related SBS4, the ROS-related SBS18 and the signature with unknown etiology, SBS16.

We did find one signature, SBS1, that showed significant differences between normal and cancer tissues. SBS1 is biased toward LRR in almost all normal tissues, and this bias is lost in cancer, both in a pan-cancer (Fig. 3A,B) and in a tissue-specific approach (Fig. 3C). Further analysis negates several possible confounders that may artificially cause such bias. First, the neutrality of SBS1 was found in different cancer projects (Fig. 3C), ruling out the possibility that the results are skewed due to one or two projects. Second, also in absolute terms there is significantly stronger bias toward LRR in normal versus cancer tissues (P < 2.2e−16, two-sided Wilcoxon rank-sum test) (Supplementary Fig. 4A,B), while this was not true for SBS5 (P = 0.23) (Supplementary Fig. 4C). This suggests that the results are not due to the relative contribution method. Lastly, the various projects use different methodologies for normal tissues acquisition^15^—laser capture microscopy was used in Colon and Hepatocytes^23,24^; Single-cell-derived colonies was used for lung bronchial epithelial cells^25^; and a combination of single cell colony with inferring mutations from both DNA and RNA was used in the Melanocytes study^26^. Receiving similar results across different tissue processing and sequencing methodologies, rules out the possibility that the results stem from the acquisition protocol.

SBS1 is a ubiquitous, clock-wise mutational signature stemming from failure to repair G:T mismatches initiated by spontaneous or enzymatic deamination of 5-methylcytosine to thymine. Thus, changes in the distribution of methylated CpG is supposed to change SBS1 distribution. Direct evaluation of the association between methylation and mutation is impossible since there is no information about the methylation status of mutated C prior to the mutation. Yet it is known that cancer transformation is accompanied by widespread DNA methylation changes. Most cancer cells exhibit a global genome-wide hypomethylation, in conjunction with a hyper methylation in CpG islands^27^. Thus we expected that in cancer cells there will be relatively more SBS1 type mutations (N[C > T]G, where N could be any nucleobase) in CpG islands and relatively less in non-island regions. Indeed, calculating the relative frequencies of SBS1 mutations across CpG islands and non-islands regions (see “Methods”) revealed a clear (P < 0.008, two-sided paired Wilcoxon rank-sum test) increase in mutation frequencies in CpG islands in cancer samples in liver and lung cancers both in ERR and LRR regions (Fig. 3D), suggesting a redistribution of methylation in the cancer samples. Thus, the change in the association of SBS1 with RT is actually explained by the redistribution of DNA methylation in cancer cells (see “Discussion”).

### Replication timing bias in germline cells

**Figure 4:**
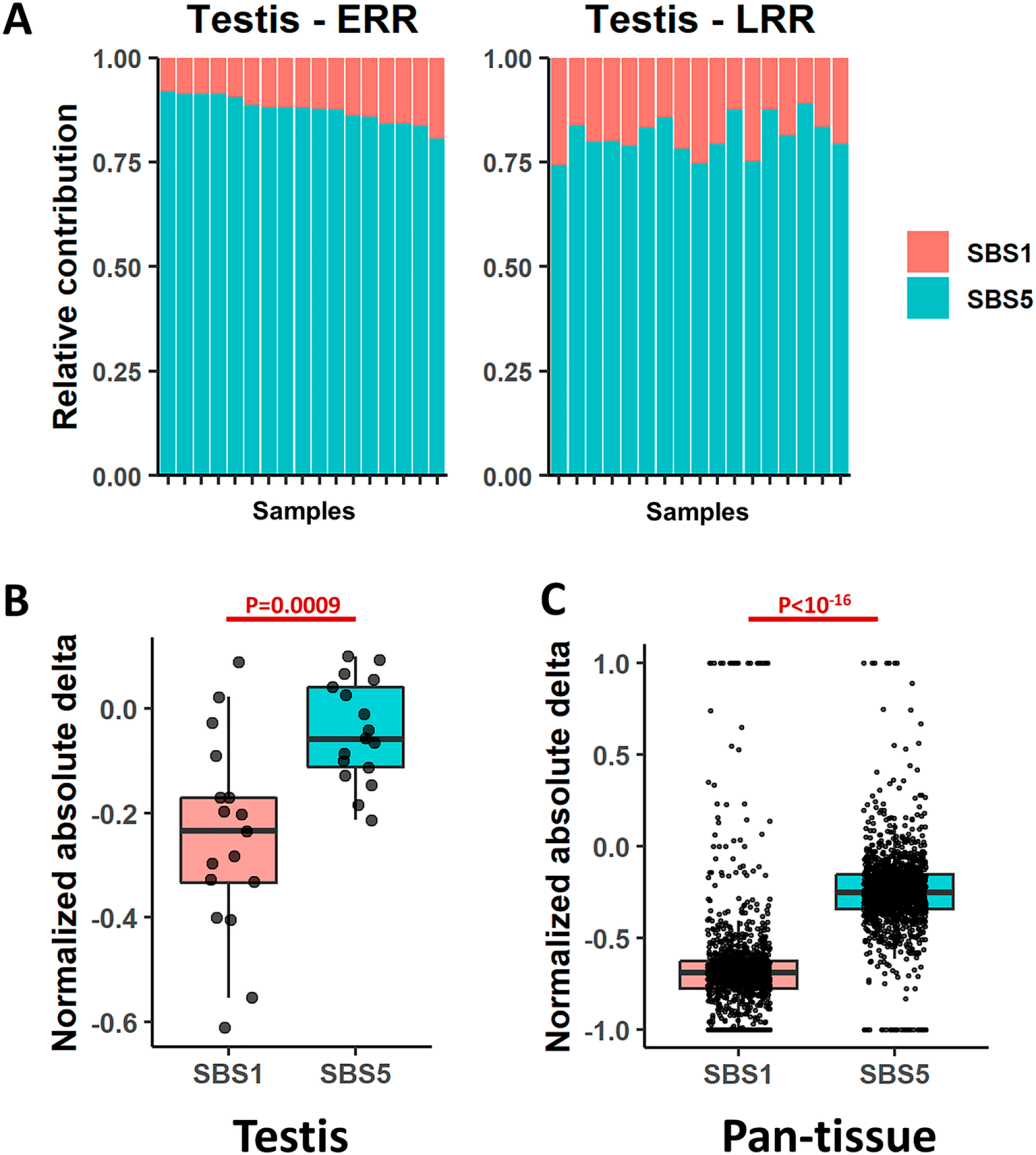
Replication timing bias in testis tissue representing mutations in germline cells. (**A**) Stacked bar plots showing the proportions of contribution of SBS1 and SBS5 in testis in ERR and LRR (left and right respectively). Samples (X axis) are ordered the same in ERR and LRR. (**B**) Boxplots showing Normalized absolute delta, i.e., $\frac{early - late}{early + late}$, in SBS1 (left) and SBS5 (right) in ERR and LRR in testis tissue. (**C**) Boxplots showing Normalized absolute delta in SBS1 (left) and SBS5 (right) in ERR and LRR, pan tissue, excluding testis. P-values derived from two-sided Wilcoxon rank-sum tests.

Moore et al. cohort provides a unique opportunity to investigate the mutational processes bias in germline cells, as they perform microdissections of seminiferous tubules which are predominantly composed of germline cells^15^. Germline cells had the lowest mutational burden across all tissues, and therefore only 37 samples passed the inclusion criteria (see “Methods”). Only SBS1 and SBS5 were found in these samples, both in ERR and in LRR. Using the measure of relative contribution, the bias of SBS1 and SBS5 was kept towards LRR and ERR respectively, as seen in the soma (Fig. 4A). Since there are only two signatures, bias in one signature will be seen as bias also in the other, as the sum is always 1. To this end, we also analyzed the bias in absolute contribution approach using normalized absolute delta (“Methods”), where we noticed that SBS1 is indeed LRR biased, with much lower normalized absolute delta than SBS5 (P = 0.0009, two-sided Wilcoxon rank-sum test) (Fig. 4B). These results are indeed similar to the distribution of SBS1 and SBS5 in other normal tissues, as seen in Fig. 4C.

## Discussion

The dissection of the association of cancer mutational processes active in different cancers with genomic features like replication timing is a very active area of research^2,3,9,18,19,28^. This also applies to the field of studying mutational processes in non-cancerous cells^14,15,29^. Yet not much was known about the changes in the activity of such processes between normal and cancerous cells. Here, we tackled this question using various data sources and computational techniques.

We found that most mutational processes that exist already in normal cells have the same association with RT (i.e., either early or late RT bias) as is found in cancerous cells (Fig. 3). This is an interesting and non-trivial observation since the association between RT and mutation rates is affected by many processes including chromatin structure, DNA repair mechanisms and replication timing itself^9,10,19^, all are vulnerable to changes during the transformation process, yet the general association remain mostly intact.

We did find that the ubiquitous SBS1 was LRR biased only in normal cells. The disappearance of the LRR bias in cancer samples is surprising since cancer cells developed from normal cells, and thus the change in SBS1’s RT bias is counter intuitive. As seen for many signatures noted above—it seems logical to expect that every mutation bias that happened in the pre-malignant stage should be maintained in the mature tumor. The difference in SBS1 can be explained by the fact that the pre-malignant mutations signatures are eclipsed in the cancer samples due to the increase in mutation rate following transformation^12^. Alternatively, we cannot rule out that cells which carry the SBS1 LRR bias are lost during positive selection and cancer evolution.

What causes the change in SBS1 bias upon cancer transformation? One possibility is changes in the distribution of methylated CpG. Indeed, we found that in liver and lung, cancer cells have more SBS1 landmark mutations in CpG islands, which are enriched in ERR (Fig. 3D). This may explain, at least partially, the disappearance of SBS1 bias toward LRR in cancer. In addition, changes in the DNA repair mechanisms may contribute to it as well. In general, base excision repair (BER) enzymes like TGD and MDB2 are needed for repairing SBS1-like mutations^30,31^. Thus, it is possible that in non-cancerous cells, we see the general tendency of repair mechanisms to be more efficient in the more accessible, ERR portion of the genome, whereas in cancer the increased mutation burden and faster replication cycles cause a less functional BER enzymes activity also in ERR, which eliminates the RT bias. Yet, such mechanisms should be more general and thus we would expect to see similar effects on other signatures which are based on failure of DNA repair mechanisms (such as APOBEC signatures). A recent study^32^ proposed a specific mechanism, which may contribute to the difference between normal and cancerous tissues. Yang et al*.*, found that the presence of a G:T mismatch increased CEBPβ binding affinity in binding sites of CCAAT/enhancer binding proteins (CEBP), which in turn inhibits the repair of such mutations. Therefore, the higher mutation load in cancer may exacerbate the inhibition of repairing such mutations both in ERR and LRR, and the RT bias diminishes. However, this cannot be the main mechanism, as only a small fraction of SBS1 mutations is derived from CCAAT sites.

Finally, data of mutations in testis cells allowed assessing the RT bias of signatures in the germline. These mutations are not affected by the process of evolutionary selection, as in the case of germline mutations databases like dbSNP^33^. Similar RT biases were found in these tissues compared to somatic tissues suggesting that in terms of RT there is no difference of the basic mechanisms responsible for the fixation of SBS1 and SBS5 related mutations.

Previous analysis of the association of mutational signatures and RT in normal cells^15^ found that most mutational signatures are more abundant in LRR. Our results differ from the analysis of Moore et al. in finding that some of the signatures are more abundant in ERR. This discrepancy may stem from the use of a larger cohort of mutations, focusing the analyses to the robust constitutive parts of the genome and using a relative rather than absolute contribution of signatures, in the current study.

There are several limitations to our study. First, it is worth mentioning that differences in occurrences of signatures in ERR vs. LRR can be affected by the sensitivity of the mutational signature’s extraction method, missing low magnitude exposure, and not solely due to regional differences^15^. However, we used one of the most accurate, robust and well-known frameworks^34^, and the results are consistent across cancer types and studies. Moreover, we performed the analyses using an additional mutational signatures algorithm, and found similar results (Supplementary Fig. 1). Second, distinguishing between a real somatic mutation and sequencing error in normal cells is challenging and few methodologies were developed to this end^15^. Since each methodology has limitations, it is possible that method-related issues affect our results and/or the comparison with results in cancer samples. However, we showed consistent results of the same phenomenon (Fig. 3C) in data from multiple studies which used 3 different methods. Moreover, few additional signatures show a milder and opposite changes upon cancer transformation. While SBS1’s bias was changed from LRR bias in normal cells to no RT bias in cancer, SBS7b, for example, is more ERR biased in normal cells than in cancer (Fig. 3A). Changes to both directions suggest that the changes stem from changes in specific repair mechanisms and do not relate to the different NGS technologies. However, we acknowledge that differences between the RT bias of normal and cancerous cells could exist, but with a lower effect size and lack of sample size large enough to statistically support them. Third, there are many signatures that are active only in cancer cells. For example, mismatch repair-related signatures like SBS6, SBS44 and more, polymerase epsilon related SBS10a/b/c/d, and BER-related SBS30 are active almost solely in cancer cells. Thus, we cannot assess how RT would impact their activity in non-cancerous cells. Finally, we analyzed the data by separating the genome to early and late replicating regions, and thus we cannot see bias of mutational signatures towards middle S phase.

Taken together, our analyses delineated the RT bias of mutational signatures in normal cells across numerous different studies, various donors, tissues and cells. Furthermore, we revealed, for the first time to our knowledge, that most of mutational processes active in non-cancerous and cancerous cells have the same RT bias to ERR or LRR respectively, except for the ubiquitous signature SBS1 which showed a striking RT bias change between cancer and non-cancerous cells.

## Methods

### Data sources

Somatic mutations in non-cancerous cells were downloaded from two main sources: (i) SomaMutDB^14^ which included data from 24 published papers, and (ii) Supplementary information from Moore et al. paper^15^. Cancer mutations were downloaded from the Pan-Caner Analysis of Whole Genomes (PCAWG)^21^.

### Replication timing annotation

We used constitutive replication timing regions as described in Ref.^9^. In brief, these regions constitute approximately 40% of the human genome that have the same RT across 26 tissues examined^35^, and thus provide a way to minimize the effect of RT variation across cell types. These regions’ RT were shown by us to be a good proxy for cancer RT across various cancers, with high correlation between the constitutive RT regions and direct whole genome RT profiling of cancer models^9^. We used the median RT to separate between early and late replicating regions. Among the constitutive RT regions, 706 Mb are defined as early replicating regions (ERR) and 583 Mb as late replicating regions (LRR).

### Mutational signatures analysis

Trinucleotide mutational profiles were extracted using SigProfilerMatrixGenerator^36^. Samples with at least 50 Single Base Substitution (SBS) events both in ERR and LRR were passed to mutational signatures analysis. Mutations in ERR and LRR were normalized according to the trinucleotide context of the whole genome. Signatures were extracted by Non-negative Matrix Factorization (NMF) using SigProfilerExtractor (v1.14) framework^37^ and decomposed using COSMIC v3.2 SBS signatures. Similarity between the original and reconstructed mutational profile of each tumor was calculated by cosine similarity, and only samples with at least 80% similarity were passed to downstream analyses.

### Replication timing bias

Somatic mutations are unevenly distributed across the genome, and late replicating regions tend to accumulate more mutations^6^. To study different mutagenesis mechanisms which attribute to this phenomenon, we extracted mutational signatures separately in ERR and LRR.

To evaluate which signature has an RT bias, and whether it is towards ERR or LRR, we used the delta metric. The delta is the relative contribution of each signature in ERR minus its relative contribution in LRR. Relative contribution of each signature in each sample was calculated as the number of mutations attributed for a specific signature in a specific sample, divided by the sum of mutations in that sample. The Normalized absolute delta measure is the absolute delta divided by the absolute sum, i.e., $\frac{early - late}{early + late}$. This approach takes into account the absolute contribution of mutations, in a scale between − 1 and 1 so a comparison between different projects is possible. Positive delta implies ERR bias (more relative/normalized absolute contribution in ERR) and negative delta implies LRR bias. A two-sided Wilcoxon ranks-sum test was used to evaluate where the differences between ERR and LRR are statistically significant.

### Comparison of normal vs cancer

Colorectal cancer (COAD), liver hepatocellular carcinoma (HCC), lung non-small cell lung cancer (NSCLC) and melanoma samples from the PCAWG were chosen to perform the comparison of mutational signatures RT bias in cancerous vs. non-cancerous cells. We chose these projects since we had sufficient normal cells matching samples, and the signatures found in those matching samples covered most of the signatures found in normal tissues. Cancer samples were processed exactly the same as the non-cancerous samples.

### CpG islands analysis

Annotations of CpG islands regions were constructed from the USCS genome browser^38^. These regions were then intersected with the ERR and LRR. To test CpG islands/non-islands mutation frequencies ratio we counted the total number of N[C > T]G mutations in each region (CpG islands in ERR; CpG islands in LRR; non-island in ERR; and non-island in LRR) and normalized it by the number of CG occurrences in the region (CpG islands-ERR: 3.7 Mb; CpG islands-LRR: 0.35 Mb; non-islands-ERR: 16.7 Mb; non-islands-LRR: 7.3 Mb).

We included in these analyses only lung and liver tissues, since in those tissues most N[C > T]G mutations are derived from SBS1. Colon/Colon cancer and Melanocytes/Melanomas were excluded from this analysis. Colon cancer is associated also with MMR and MMR related signatures—SBS6, SBS15 and SBS44, which are also characterized by N[C > T]G mutations^22^, and thus cannot be distinguished from SBS1 mutations. Melanocytes samples contain almost no N[C > T]G mutations.

### Statistics

Statistical analyses were performed using R version 4.1.0. If not stated otherwise, the comparison of two distributions of continuous values was tested with a Wilcoxon rank sum test. For multiple comparisons, P-values were corrected by false discovery rate (FDR). All box plots are presented according to the standard box plot notation in R (ggplot2 package).

## Supplementary Information

Supplementary Figures.

## Data Availability

All datasets analyzed during the current study are publicly available as described in the “Methods” section. Somatic mutations in non-cancerous cells were downloaded from SomaMutDB (https://vijglab.einsteinmed.org/SomaMutDB/) and from the Supplementary information of Moore et al. paper^15^. Cancerous somatic mutations of the PCAWG were downloaded from https://dcc.icgc.org/pcawg.
