# Supplementary material for: Mutational signatures association with replication timing in normal cells reveals similarities and differences with matched cancer tissues
Source: Sci Rep. 2023 May 15;13:7833. doi: 10.1038/s41598-023-34631-9 (PMC10185532; doi:10.1038/s41598-023-34631-9)
Supplement: Supplementary file 1 — Supplementary Figures. [file 41598_2023_34631_MOESM1_ESM.pdf]

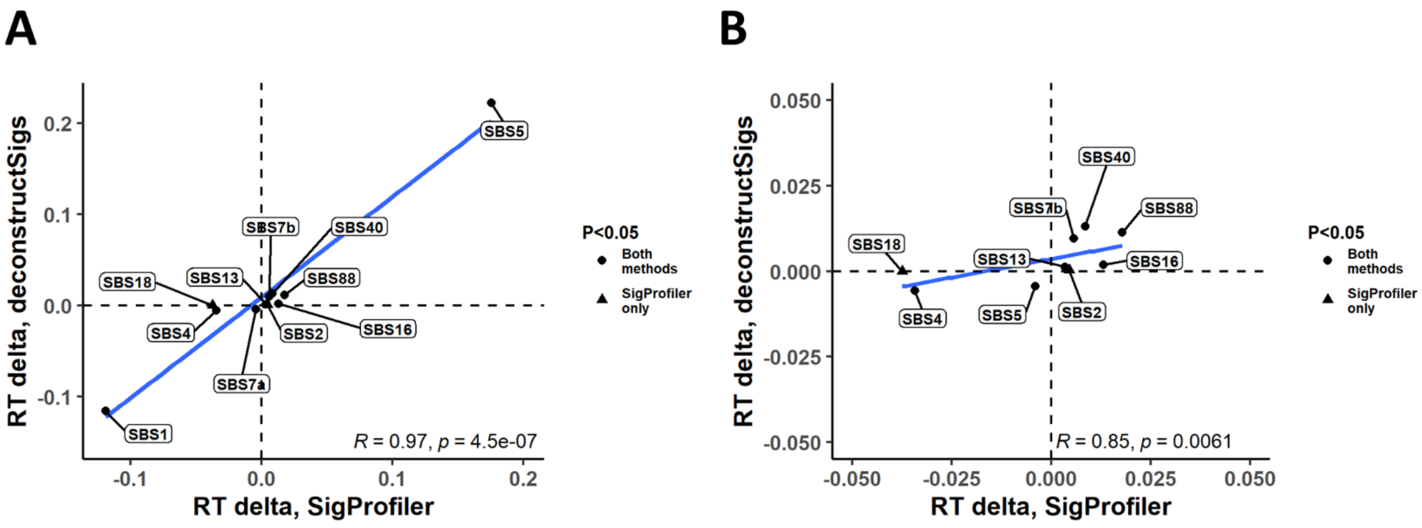

Supplementary Figure 1 | Correlation between SigProfiler and deconstructSigs frameworks. A) Correlation of main signatures which showed RT bias in a pan-tissue manner or in colon, liver, and lung tissues. P-values derived from a two-sided Wilcoxon rank-sum test. **B)** Zoom-in of A, showing all signatures except SBS1 and SBS5. The correlation here is weaker, and two signatures (SBS18 and SBS2) are statistically significant RT-biased only using SigProfiler. However, since deconstructSigs tended to assign less contribution to most signatures except SBS1 and SBS5, it is likely that deconstructSigs need a larger sample size in order to achieve significance.

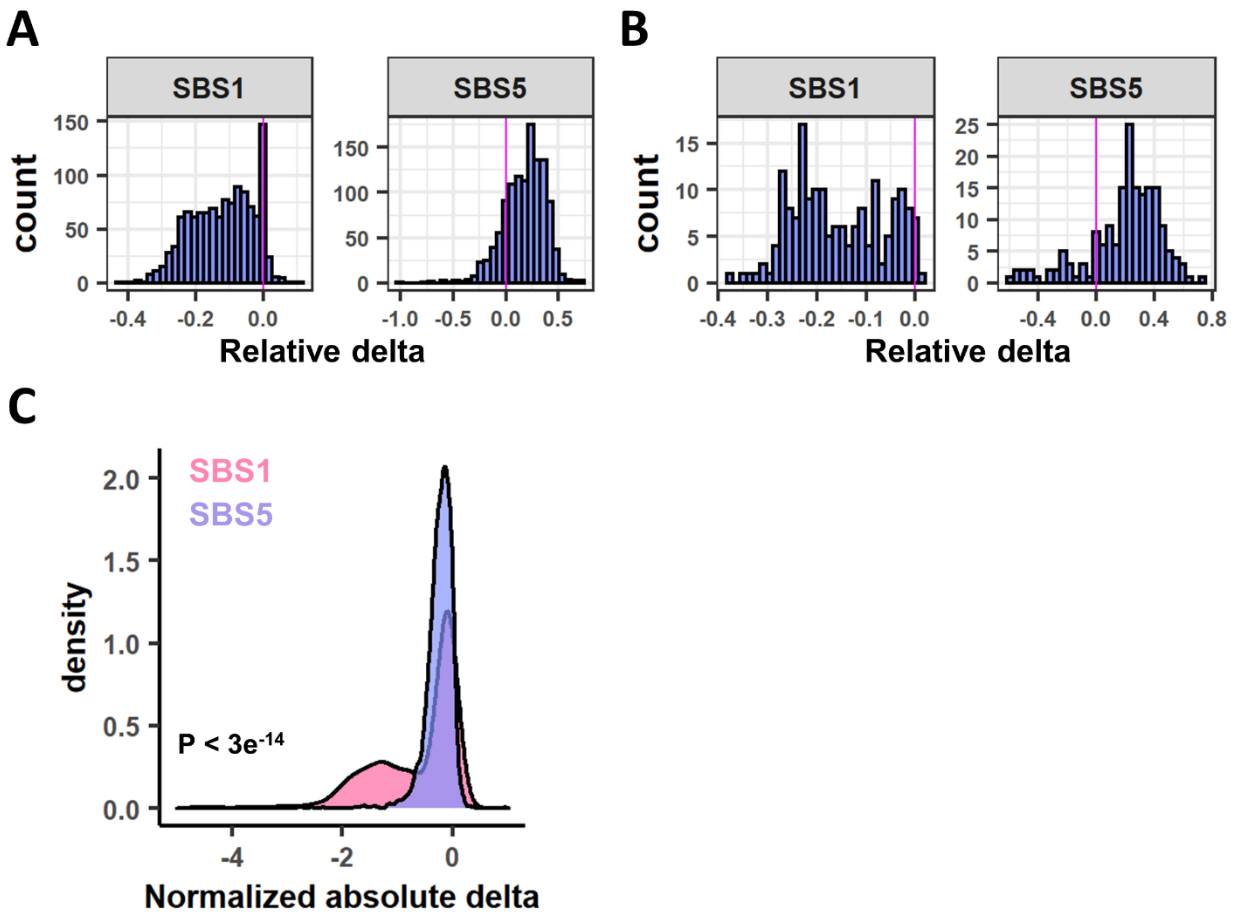

Supplementary Figure 2 | RT bias distributions of SBS1 and SBS5 in a pan-tissue approach. A) Histograms of relative RT delta ($early - late$) in SBS1 (left) and SBS5 (right) across all Mixed cohort. **B)** Histograms of relative RT delta in SBS1 (left) and SBS5 (right) across all Moore et al. cohort. **C)** Density plots of normalized absolute delta, i.e., $\frac{early - late}{early + late}$, of SBS1 and SBS5. P-value derived from a two-sided Wilcoxon rank-sum test.

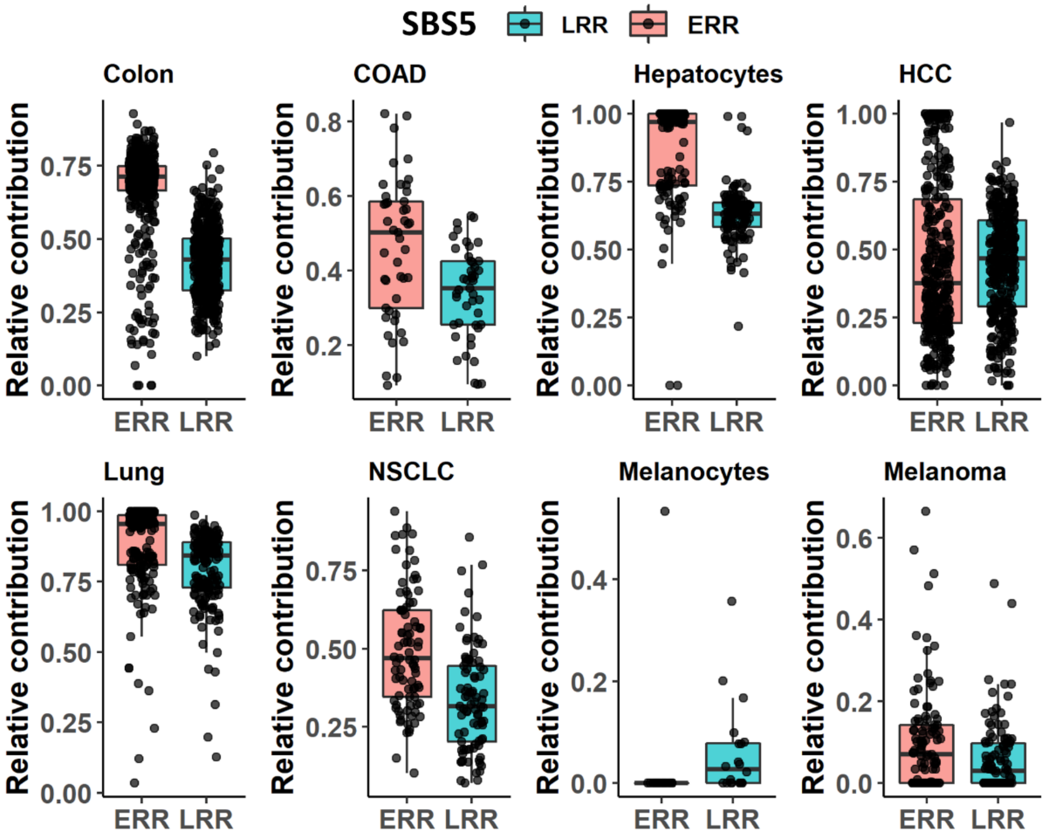

Supplementary Figure 3 | SBS5's contribution comparison in normal and cancer tissues. Boxplots showing the contribution of SBS5 in ERR and LRR in a normal tissue compared to cancer tissue. Left upper, non-cancer colon cells (left) and colon adenocarcinoma samples (right); Right upper, hepatocytes (left) and Hepatocellular carcinoma (right); Left lower, Lung (left) and NSCLC (right); Right lower, melanocytes (left) and melanoma (right). NSCLC, non-small cell lung cancer.

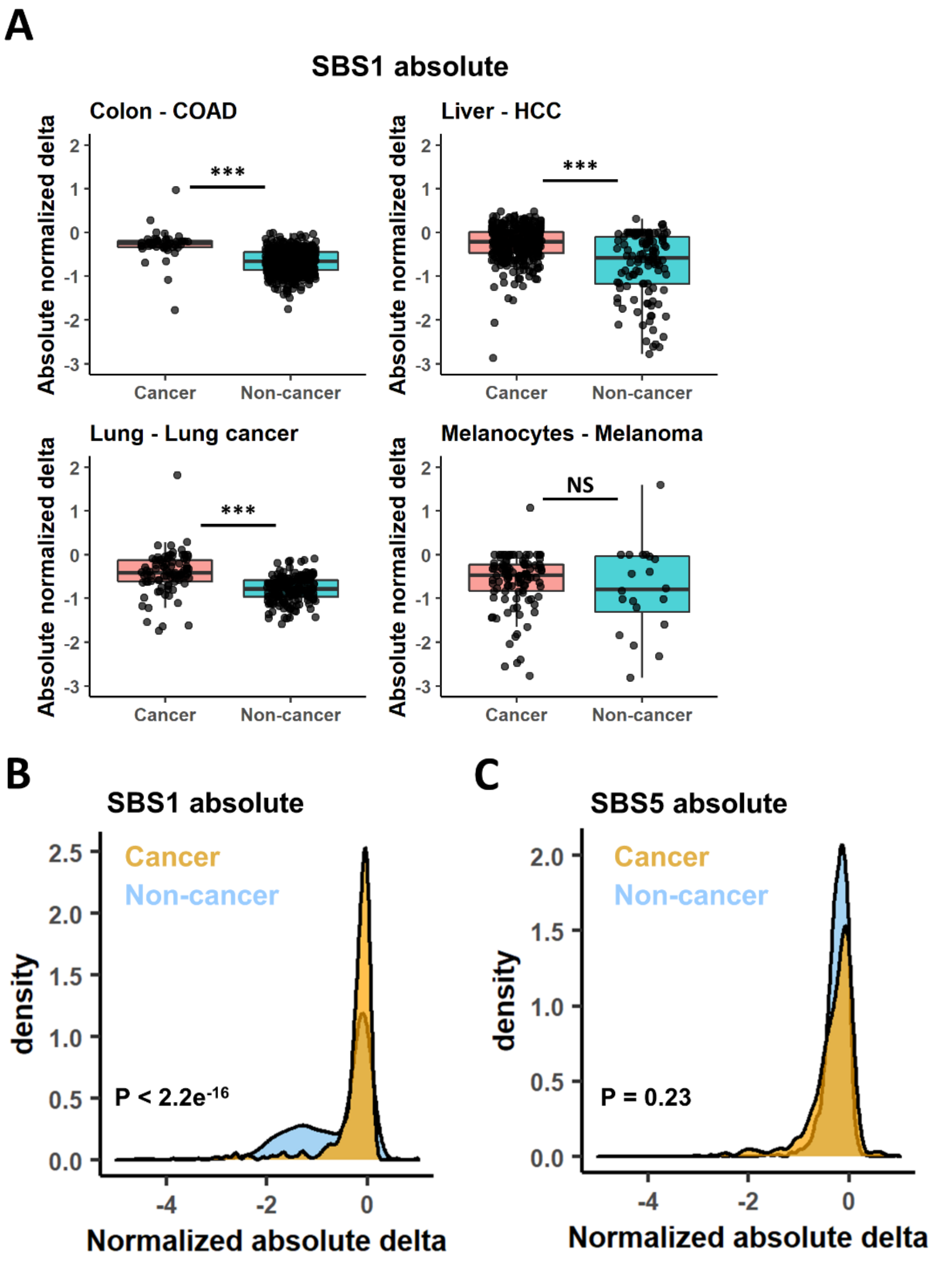

Supplementary Figure 4 | LRR bias of SBS1 in normal vs. cancer tissues, absolute contribution. A) Boxplots showing the normalized absolute delta, i.e., $\frac{early - late}{early + late}$, in cancerous vs. non-cancerous cells in matching tissue-cancer projects. P-values derived from two-sided Wilcoxon rank-sum tests. Left upper, colon and colorectal tissues; Right upper, liver and hepatocellular carcinoma; Left lower, lung and non-small cell lung cancer; Right lower, melanocytes and melanoma. **B)** Density plot of the normalized absolute delta of SBS1 in normal and cancer tissues. P-value derived from a two-sided Wilcoxon rank-sum test. **C)** Density plot of the normalized absolute delta of SBS5 in normal and cancer tissues. P-value derived from a two-sided Wilcoxon rank-sum test.
